# A210 THE BURDEN OF IBD HOSPITALIZATION IN CANADA: AN ASSESSMENT OF THE CURRENT AND FUTURE BURDEN IN A NATION-WIDE ANALYSIS

**DOI:** 10.1093/jcag/gwac036.210

**Published:** 2023-03-07

**Authors:** S Coward, E I Benchimol, C Bernstein, J A Avina-Zubieta, A Bitton, L Hracs, J Jones, E Kuenzig, L Lu, S K Murthy, Z Nugent, A R Otley, R Panaccione, J -N Pena-Sanchez, H Singh, L E Targownik, J W Windsor, G Kaplan

**Affiliations:** 1 University of Calgary, Calgary; 2 The Hospital for Sick Children, Toronto; 3 University of Manitoba, Winnipeg; 4 University of British Columbia, Vancouver; 5 McGill University, Montreal; 6 Dalhousie University, Halifax; 7 Arthritis Research Canada, Vancouver; 8 The Ottawa Hospital, Ottawa; 9 University of Saskatchewan, Saskatoon; 10 University of Toronto, Toronto, Canada

## Abstract

**Background:**

Hospitalizations pose a significant burden on both the individual and the healthcare system. Those with inflammatory bowel disease (IBD) are at increased risk of hospitalization as compared to the general population due to flaring of disease activity and complications related to IBD. The advent of biologics over the past twenty years may have influenced the rates of hospitalization for IBD.

**Purpose:**

To assess current and forecast the overall hospitalization rates of those with IBD stratified by types of hospitalizations (all cause hospitalizations, IBD-related, and IBD-specific).

**Method:**

Population-based administrative data on hospitalization of IBD (2002-2014) were obtained from: AB, BC, MB, and SK. Data were age and sex standardized to the matching year and aggregated into a representative sample of the Canadian population. Hospitalization rates were assessed as follows: 1. All cause hospitalizations: all admissions regardless of indication; 2. IBD-specific: an admission directly resulting from IBD (e.g., IBD-flare); 3. IBD-related: an admission for IBD, or a symptom or comorbidity associated with IBD (e.g. rheumatoid arthritis). Using prevalence estimates from the provinces, hospitalization rates (per 100 persons with IBD) were calculated, with 95% confidence intervals (CI). Autoregressive Integrated Moving Average models were created to estimate number of hospitalizations and corresponding prevalence to forecast hospitalization rates to 2030 with 95% prediction intervals (PI). Poisson (or negative binomial) regression estimated the Average Annual Percentage Change (AAPC), with 95% CIs, of the forecasted data.

**Result(s):**

In 2002 there were 35.3 per 100 (95%CI: 34.7, 35.9) all cause hospitalizations for IBD patients and this decreased to 24.9 per 100 (24.5, 25.2) in 2014. Similar trends were seen for IBD-specific hospitalizations [16.8 per 100 (95%CI: 16.4, 17.2) in 2002 to 8.7 per 100 (95%CI: 8.5, 9.0) in 2014] and IBD-related (22.6 per 100 (95%CI: 22.1, 23.1) in 2002 to 13.4 per 100 (95%CI: 13.2, 13.7) in 2014). When forecasted out to 2030 all hospitalization types were significantly decreasing—the AAPC for all cause hospitalizations was -2.12% (95%CI: -2.31, -1.93), -3.77% (95%CI: -4.63, -3.08) for IBD-specific, and -3.09% (95%CI: -3.65, -2.62) for IBD-related. By 2030, the rates of hospitalization are forecasted to be 17.0 per 100 (95%PI: 16.2, 17.9), 4.6 per 100 (95%PI: 3.7, 5.4), and 7.9 per 100 (95%PI: 6.9, 8.9) for all cause, IBD-specific, and IBD-related, respectively.

**Image:**

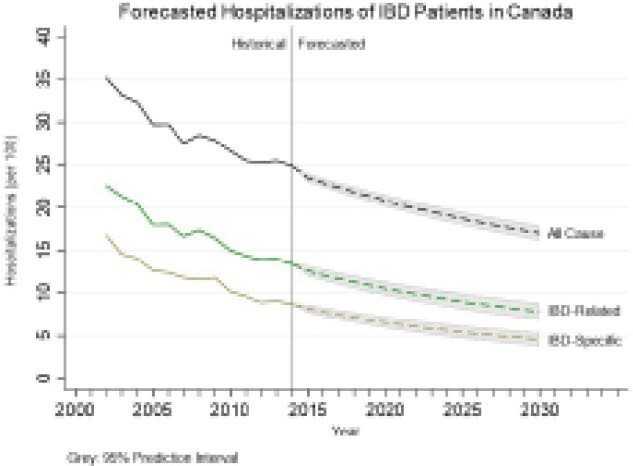

**Conclusion(s):**

In Canada, rates of hospitalizations for those with IBD have decreased from 2002 to 2014. The use of anti-TNF therapy in conjunction with the evolution of clinical monitoring, management and guidelines, likely has contributed to dropping hospitalization rates. Forecast models estimate a continued drop in hospitalization rates out to 2030. Importantly, healthcare resource planning should account for the shift from hospital-based to clinic-centric models of IBD care.

**Please acknowledge all funding agencies by checking the applicable boxes below:**

CIHR

**Disclosure of Interest:**

S. Coward: None Declared, E. Benchimol Consultant of: Hoffman La-Roche Limited and Peabody & Arnold LLP for matters unrelated to medications used to treat inflammatory bowel disease and McKesson Canada and the Dairy Farmers of Ontario for matters unrelated to medications used to treat inflammatory bowel disease., C. Bernstein Grant / Research support from: Unrestricted educational grants from Abbvie Canada, Janssen Canada, Pfizer Canada, Bristol Myers Squibb Canada, and Takeda Canada. Has received research grants from Abbvie Canada, Amgen Canada, Pfizer Canada, and Sandoz Canada and contract grants from Janssen, Abbvie and Pfizer, Consultant of: Abbvie Canada, Amgen Canada, Bristol Myers Squibb Canada, JAMP Pharmaceuticals, Janssen Canada, Pfizer Canada, Sandoz Canada, and Takeda., Speakers bureau of: Abbvie Canada, Janssen Canada, Pfizer Canada and Takeda Canada, J. A. Avina-Zubieta: None Declared, A. Bitton: None Declared, L. Hracs: None Declared, J. Jones Consultant of: Janssen, Abbvie, Pfizer, Takeda, Speakers bureau of: Janssen, Abbvie, Pfizer, Takeda, E. Kuenzig: None Declared, L. Lu: None Declared, S. Murthy: None Declared, Z. Nugent: None Declared, A. Otley Grant / Research support from: Unrestricted educational grants from AbbVie Canada and Janssen Canada, Consultant of: Advisory boards of AbbVie Canada, Janssen Canada and Nestle, R. Panaccione Consultant of: Abbott, AbbVie, Alimentiv (formerly Robarts), Amgen, Arena Pharmaceuticals, AstraZeneca, Biogen, Boehringer Ingelheim, Bristol-Myers Squibb, Celgene, Celltrion, Cosmos Pharmaceuticals, Eisai, Elan, Eli Lilly, Ferring, Galapagos, Fresenius Kabi, Genentech, Gilead Sciences, Glaxo-Smith Kline, JAMP Bio, Janssen, Merck, Mylan, Novartis, Oppilan Pharma, Organon, Pandion Pharma, Pendopharm, Pfizer, Progenity, Protagonist Therapeutics, Roche, Sandoz, Satisfai Health, Shire, Sublimity Therapeutics, Takeda Pharmaceuticals, Theravance Biopharma, Trellus, Viatris, UCB. Advisory Boards for: AbbVie, Alimentiv (formerly Robarts), Amgen, Arena Pharmaceuticals, AstraZeneca, Biogen, Boehringer Ingelheim, Bristol-Myers Squibb, Celgene, Eli Lilly, Ferring, Fresenius Kabi, Genentech, Gilead Sciences, Glaxo-Smith Kline, JAMP Bio, Janssen, Merck, Mylan, Novartis, Oppilan Pharma, Organon, Pandion Pharma, Pfizer, Progenity, Protagonist Therapeutics, Roche, Sandoz Shire, Sublimity Therapeutics, Takeda Pharmaceuticals, Speakers bureau of: AbbVie, Amgen, Arena Pharmaceuticals, Bristol-Myers Squibb, Celgene, Eli Lilly, Ferring, Fresenius Kabi, Gilead Sciences, Janssen, Merck, Organon, Pfizer, Roche, Sandoz, Shire, Takeda Pharmaceuticals, J.-N. Pena-Sanchez: None Declared, H. Singh Consultant of: Pendopharm, Amgen Canada, Bristol Myers Squibb Canada, Roche Canada, Sandoz Canada, Takeda Canada, and Guardant Health, Inc.,, L. Targownik Grant / Research support from: Investigator initiated funding from Janssen Canada, Consultant of: [Advisory board] AbbVie Canada, Takeda Canada, Merck Canada, Pfizer Canada, Janssen Canada, Roche Canada, and Sandoz Canada, J. Windsor: None Declared, G. Kaplan Grant / Research support from: Ferring, Janssen, AbbVie, GlaxoSmith Kline, Merck, and Shire, Consultant of: Gilead, Speakers bureau of: AbbVie, Janssen, Pfizer, Amgen, and Takeda

